# A Retrospective Study with a Commercial Vaccine against Lyme Borreliosis in Dogs Using Two Different Vaccination Schedules: Characterization of the Humoral Immune Response

**DOI:** 10.3390/vaccines11010043

**Published:** 2022-12-25

**Authors:** Claudia K. Wilczek, Jasmin Wenderlein, Stephanie Hiereth, Reinhard K. Straubinger

**Affiliations:** Chair of Bacteriology and Mycology, Institute of Infectious Diseases and Zoonosis, Department of Veterinary Sciences, Faculty of Veterinary Medicine, LMU Munich, Veterinärstr. 13, 80539 Munich, Germany

**Keywords:** Lyme borreliosis, *Borrelia burgdorferi* sensu stricto, *Borrelia garinii*, *Borrelia afzelii*, vaccination, OspA, vaccination scheme, booster, *Ixodes ricinus*

## Abstract

Lyme borreliosis, a multisystemic disease caused by spirochetes of the genus *Borrelia*, is the most common tick-borne disease in the northern hemisphere. Differently from human medicine, several vaccines are available for dogs. To provide the best protection possible, vaccination schemes should be adapted regularly to meet the needs resulting from an increased tick exposure risk due to an inescapable climate change. In this retrospective study, a total of 183 vaccinations were performed with a commercial, multivalent vaccine against Lyme borreliosis, and vaccinated dogs were monitored over an observation period of 13 months. Dogs were either vaccinated on days 0 and 21 and a booster on day 365 (standard vaccination schedule), or with an additional booster vaccination on day 180. Canine serum samples were then tested for their borrelia-specific antibody levels using a two-tiered test system consisting of a kinetic ELISA followed by a line immunoassay. Dogs vaccinated with the standard vaccination schedule displayed decreasing antibody levels between days 120 and 360, which is probably insufficient to prevent an infection with borreliae. In contrast, the additional booster vaccination received on day 180 intercepts this decline in antibody levels between days 225 and 360, providing a sufficient immunity to prevent infection. The results from this retrospective study allow us to recommend a basic vaccination schedule with an additional booster vaccination on day 180 to ensure the best possible protection for dogs against Lyme borreliosis.

## 1. Introduction

*Ixodes ricinus* is the most common tick species in Europe feeding on mammals (e.g., mice, rabbits, dogs, cats, deer, humans) and birds [1]. Beside bacteria from the *Borrelia burgdorferi* sensu lato (*Bb*sl) complex causing Lyme borreliosis (LB), *I. ricinus* ticks transmit pathogens like *Anaplasma* spp., *Ehrlichia* spp., and tick-borne encephalitis virus. LB is the most common tick-borne disease (TBD) in the northern hemisphere [2] displaying high geographical differences in its species distribution between the continents. The most important species in Europe are *B. afzelii*, *B. garinii*, and *B. burgdorferi* sensu stricto (*Bb*ss) [3].

The ticks’ questing activity is dependent on the temperature. Ticks start questing at a temperature of at least 7 °C [4,5]. Due to climate change and an estimated temperature increase of 0.9–2.3 °C by 2100—when climate policy is as ambitious in its actions as pledged by politicians [6]—we must assume, that tick reproduction and activity will increase over time, and ticks will populate habitats that they could not inhabit until recently, e.g., higher altitudes [7,8] or regions closer to the arctic circle [9]. For Sweden, a connection between an increase in TBD and climate change has already been discovered in 2001 [10]. Here, an increase in TBD incidence was associated with mild winters, temperatures favoring spring development, long autumns in the previous year, temperatures favorable for ticks’ questing activity early in the year, and a deeper snow cover [1,10]. In 2007, tick activity in the winter associated with host quest has been described for the first time in Germany [11]. Therefore, dog owners must be aware of the year-round risk of tick attachment and TBD transmission. However, unlike in humans LB in dogs shows no obvious early clinical signs such as an *erythema migrans* or flu-like signs [12,13]. Nevertheless, it has been shown, that experimentally infected dogs develop clinical signs consistent with polyarthritis like joint pain, lethargy, transient fever, anorexia, and neurological alterations [12,14,15]. In Germany, the seroprevalence in dogs for *Bb*sl ranges between around 2 and 20% [16,17], though, not every infected dog displays any or noticeable clinical signs [18,19,20]. Due to the lack of early and specific clinical signs, the detection of LB is not trivial. As the cultivation and detection of *Borrelia* spp. in tissue samples or body fluids is difficult [14,21], LB is diagnosed by serologic testing via a two-tiered test consisting of a kinetic antibody ELISA (KELA) and a line immunoassay (LIA) [14,22]. However, seropositivity is not evident for LB and the diagnosis should be based on known tick exposure in an endemic region, compatible clinical signs, and laboratory findings.

The prevention of LB is based on three columns: tick removal [23], application of repellents [24] or ectoparasiticides [25], and vaccination [26]. Since many decades, vaccinations are playing an important role in protecting humans and animals against diseases, and vaccination of dogs against LB will become an even more important tool for LB prevention as more ticks develop resistance against acaricides [27].

Currently, in Germany the vaccination of dogs against LB is based on immunization with lysate vaccines containing a special lipoprotein known as outer surface protein A (OspA) [28]. This antigen enables the vaccinees to produce antibodies against OspA. Interestingly, OspA is expressed only by *Bb*sl organisms in the tick and in vitro cultures [29,30,31]. During the ticks’ blood meal, the mammalian blood with antibodies against the OspA will be sucked into the ticks’ intestine where the OspA on the spirochetes’ surface will bind the antibodies. In turn, the bound antibodies immobilize the spirochetes and prevent the infection of the mammalian host [29,32]. In the 1990s, a LB vaccine with the OspA for the vaccination of humans with an efficiency of 76% was introduced in the US market. However, low public demand and acceptance directed the manufacturers to remove this vaccine from the market in 2002 [33,34,35]. Meanwhile, there is an increasing interest to introduce again a human LB vaccine since clinical trials are conducted (NCT05477524). In 2022, three vaccines against LB for dogs targeting different species of the *Bb*sl-complex are authorized for use in Germany. The choice of borrelial species or antigens present in the vaccine should be adapted to the geographical diversity of the *Borrelia* spp. prevailing in the region, where the vaccine will be used [36,37], resulting in the notion that a specific LB vaccine cannot be used worldwide. As described above, vaccines applied in Germany should protect against *Bb*ss, *B. garinii*, and *B. afzelii*.

Protection against disease and—more desirable—against infection is not only affected by the antigens present in the vaccine but is highly dependent on the vaccination schedule applied. In the case of LB most veterinarians adhere to a simple scheme based on a basic immunization consisting of two initial injections two to three weeks apart followed by yearly revaccinations. Yet, thorough analyses of antibody levels in humans after basic vaccination have shown, that protective antibody levels wane rapidly, and protection is lost within six months after vaccination was started [38]. This might be the case in dogs as well [17], however, the vaccine used in this study has not been tested previously. The vaccination schedule with a booster vaccination after six months seems reasonable, as there is evidence also in horses [39] that an additional booster vaccination after six months is advisable as antibodies against OspA seem to gradually lower after time, resulting in a possible gap in immunity [17,39,40]. As shown in a study in dogs using vaccines protecting against one or two *Bb*sl-complex species, a booster vaccination a few weeks after the basic immunization increased antibody levels [17]. However, vaccines protecting against a single or two *Bb*sl-complex species display no or at best a low protective effect against other borrelial species [17]. To the best of our knowledge, the effect on the humoral IgG OspA response in association with the vaccination schedule of a lysate LB vaccine for dogs using three *Bb*sl-complex species has not been analysed. Therefore, this study aimed to characterize and compare IgG antibody levels arising from two different vaccination schedules using a polyvalent vaccine (Merilym 3, Boehringer Ingelheim Vetmedica GmbH, Ingelheim am Rhein, Germany). For this purpose, dogs’ IgG antibody levels were assessed quantitatively and semi-quantitatively using the two-tiered-test consisting of a KELA and a LIA. Further, a species-specific rOspA KELA was performed to measure the dogs’ immune response against *Bb*ss, *B. garinii*, and *B. afzelii*.

## 2. Materials and Methods

The ethic commission of the veterinary faculty of the LMU Munich approved the use of canine serum samples in the proposed study (reference number 327-20-09-2022).

The study was conducted in a veterinary practice in Amberg (Upper Palatinate, Bavaria, Germany) receiving dog patients from the city of Amberg with a low LB risk of 23.75 reported human cases (RHC) per 100,000 inhabitants (I), the district Amberg-Sulzbach with a medium-high LB risk of 201.95 RHC per 100,000 I, and the districts Schwandorf (292.48 RHC/100,000 I), and Neumarkt in der Oberpfalz (125.14 RHC/100,000 I) [41]. Due to the medium-high risk of human LB in the districts of Amberg-Sulzbach, Schwandorf, and Neumarkt in der Oberpfalz and many cases of tick attachment to dogs leading to anaplasmosis and LB in the region of this veterinary practice, many dog owners are willing to vaccinate their companion animals against LB, resulting in a high vaccination rate in this district. Therefore, this practice was especially suited to study the effectiveness of vaccination schedules retrospectively in an animal-experiment-free setting.

Owners asking for a LB vaccination in the practice were given the choice to either vaccinate their dogs with the standard vaccination (i.e., vaccination on days 0, 21, and 365) or to vaccinate with an additional booster (i.e., vaccination days 0, 21, 180, and 365). All dogs were tested before the first vaccination for antibodies specific to LB infection with the commercially available SNAP 4Dx Plus test (IDEXX Laboratories Inc., Westbrook, ME, USA) conducted according to the manufacturer’s instructions to rule out an active infection at the time of vaccination (i.e., day 0). An active infection would have made the treatment of dogs with antibiotics necessary before vaccination. The immunization of dogs who were tested negative in the SNAP 4Dx Plus test was then conducted using the vaccine Merilym 3 (Boehringer Ingelheim Vetmedica GmbH, Ingelheim am Rhein, Germany), which was approved for its use in dogs in March 2013 by the Paul-Ehrlich-Institut (PEI) in Lagen, Germany (PEI.V.11652.01.1). A 1.0-mL dose of this lysate vaccine contains the inactivated *Bb*sl-complex species *B. garinii*, *B. afzelii*, and *Bb*ss. As a preservative, 0.5 mg of formaldehyde are incorporated, and aluminum hydroxide serves as an adjuvant. The vaccination of the dogs was performed subcutaneously in the dorsolateral thoracal region using 24-gauge needles (0.55 mm × 25 mm; B. Braun SE, Melsungen, Germany).

When blood from the vaccinated dogs was collected for diagnostic purposes in the veterinary practice and leftovers were available, these leftover blood samples were stored upright at room temperature for 60 to 180 min until complete coagulation. Samples were then centrifuged at 4000× *g* for ten minutes. Afterward, the serum was transferred into screw cap microtubes (Sarstedt AG & Co. KG, Nümbrecht, Germany), which were labeled and stored at −20 °C until serological analysis. Blood was collected by either puncturing the right *Vena cephalica antebrachii* or the right *V. saphena lateralis* with a 20-gauge needle (0.90 mm × 40 mm; B. Braun SE) and 9-mL Serum-Monovettes Z-Gel (Sarstedt AG & Co. KG). All owners of vaccinated dogs whose blood was collected for diagnostic purposes and residuals were available for LB antibody testing agreed to the transmission and serologic analysis of their dogs’ blood at the Chair of Bacteriology and Mycology of LMU Munich. Serum samples were assigned to nine different periods according to the time that passed between the first vaccination and the sampling for diagnostic purposes. Following one year after vaccination, we divided the observation period into nine time frames and accordingly assigned them to nine descriptive time points (Table 1).

At the end of the observation period, all serum samples were tested at the Chair of Bacteriology and Mycology of LMU Munich. Serum samples were therefore thawed at room temperature. For quantitative analysis, total antibody levels specific for LB organisms were evaluated using a KELA as described elsewhere [12,17,38]. Representative samples for every time point from each study group, of which a sufficient volume was available after the first round of serological testing, were assessed for the presence of species-specific rOspA antibodies using a KELA. As the lowest number of serum samples available for one observation timepoint was seven serum samples, we used this number of samples in all KELAs to allow compatibility. Therefore, 96-well-plates were prepared with 1.0 µg of purified recombinant OspA from the strains *Bb*ss ZS7, *B. garinii* ZQ1, or *B. afzelii* PKo kindly provided by Prof. Dr. Kraiczy (Institute for Medical Microbiology and Infection Control, Frankfurt, Germany). To confirm positive results and differentiate infected from vaccinated dogs, a line immunoassay (LIA) was performed. Here, the Borrelia Veterinär plus OspA LINE (VIROTECH Diagnostics GmbH, Dietzenbach, Germany) was used to analyze and evaluate the presence of *B. burgdorferi* sensu lato-specific antibodies according to the manufacturer’s instructions.

### Statistical Analysis

Antibody levels for each dog were recorded, summarized according to the experimental groups, and compared statistically. KELA curves were visualized using OriginPro, Version 2022 (OriginLab Corporation, Northampton, MA, USA). To compare the KELA levels (i.e., IgG and species-specific) from both groups, we calculated the area under the curve (AUC) between the time points t0 and t30, t30 and t60, t60 and t120, t120 and t180, t180 and t230, t230 and t300, t300 and t350, and t350 and t390. The AUC was then tested for standard deviation using the Shapiro–Wilk test. Besides the AUC between t0 and t30, all AUCs were normally distributed. Significance for the AUC between t0 and t30 was then calculated using the Mann–Whitney test, while all other time frames were tested with the *t*-test. All analyses were conducted with OriginPro, Version 2022 (OriginLab Corporation). Significance was assumed when the *p* value was *p* ≤ 0.05.

## 3. Results

**Figure 1:**
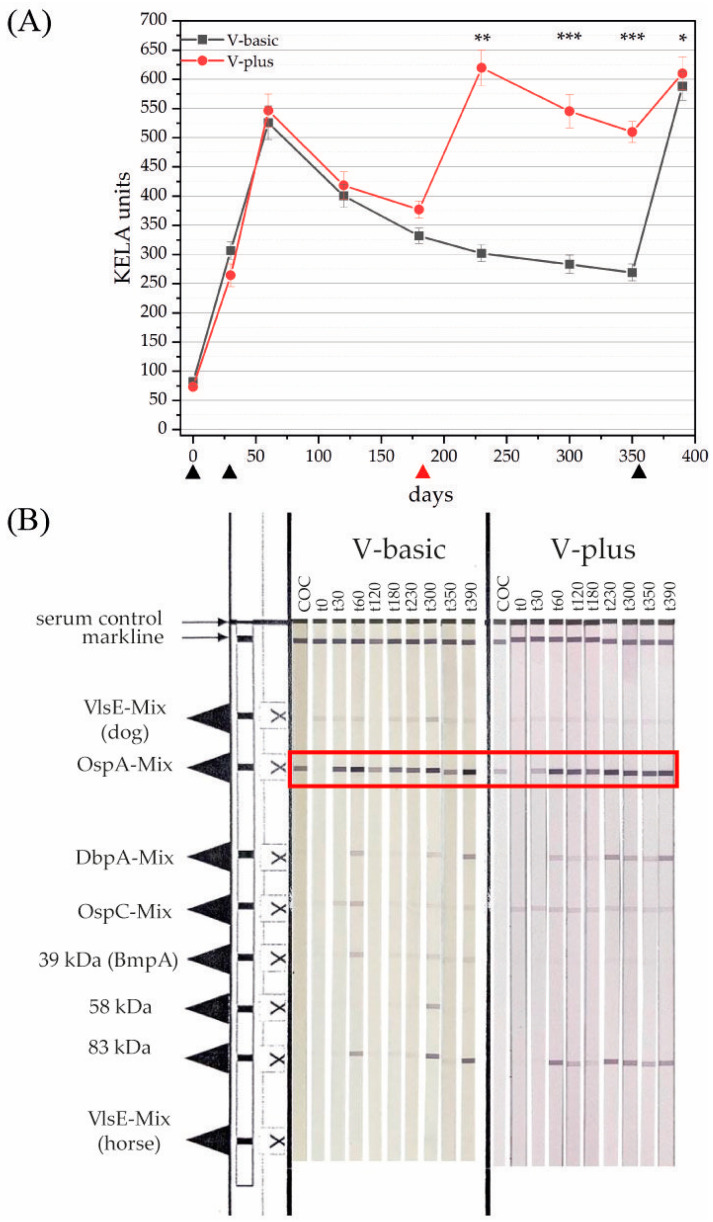
(**A**) *Borrelia burgdorferi* sensu lato-specific antibody levels in dog sera detected by KELA throughout the complete observation period. V-basic (vaccinated on days t0, t30, and t350) displayed as black squares; V-plus (vaccinated on days t0, t30, t180, and t350) shown as red circles. The whiskers depict the standard deviation at a specific time point. Black triangles show vaccination time points in both groups, while the red triangle indicates the additional booster vaccination in V-plus. (**B**) LIA strips for representative dogs from V-basic (left side) and from V-plus (right side) displaying semi-quantitative antibody reactions throughout the observational period. The first LIA strip shows the COC, which is used to determine the degree of color reaction intensity of each antigen line. Following right are the strips incubated with canine sera representative for two vacination groups in chronological order from t0 to t390. The vaccination-specific antigen signal on the LIA strips is the OspA-Mix (red box). * *p* ≤ 0.05; ** *p* ≤ 0.001; *** *p* ≤ 0.0001.

In total, 183 vaccinations against LB were performed during the project. Of these vaccinations for V-basic, 222 serum samples were analyzed for the classified time points, while for V-plus 223 serum samples were collected and examined for their antibody contents. Of these serum samples, 41% originated from female dogs, while the remaining 59% were from male dogs, 39% of the experimentees were spayed or neutered. The age of the dogs ranged between four months and eleven years. Irrespective of the vaccination group, only moderate side effects were reported. In the test population, 4% of the dogs seemed to be tired one or two days after the immunization, 2% showed signs of moderate pain, and in 0.6% moderate swelling at the injection site was reported. Serum samples displayed negative results in the SNAP 4Dx Plus before the vaccination on t0. However, when analyzing these sera from t0 with a KELA, some of these sera showed antibody levels higher than 100 (*n* = 10), which is thought to be a threshold that non-infected dogs rarely pass. When analyzing these serum samples on a LIA, a color reaction specific to infection was observed in four serum samples. Sera, which tested negative on the SNAP-test and showed antibody levels below 100 KELA units, displayed a slight color- reaction for the VlsE antigen (VlsE AG line) fainter than the cut-off control (COC) or no reaction at all on the LIA strip (Figure 1B). This is considered negative for LB infection.

The first vaccination with the vaccine was performed on t0. All samples available on t30 showed increasing antibody levels in the KELA (Figure 1A) and specific reactions to the OspA antigen on the LIA (Figure 1B). Dogs were then vaccinated a second time on t30, finalizing the basic immunization. After the second immunization, antibody levels measured with the KELA increased further until t60. At this time point, LIA strips displayed color reactions to the OspA antigen that were stronger than the COC in both groups. Color reactions of antigens beside the VlsE and stronger than the COC that occurred on the LIA strips of vaccinated canines are considered reactions to the various antigens provided with the lysate vaccine. After t60, both groups displayed a steady and clear decrease in antibody levels observable until t180 (Figure 1A); on matching LIA strips this can be observed as well as decreasing color intensity of the OspA antigen lines (Figure 1B). Dogs in V-plus, which had received an additional booster vaccination on t180, developed significantly higher antibody levels on t230 compared to dogs in V-basic (Figure 1A). The antibodies reached an average of around 600 KELA units in V-plus, compared to an average of around 300 KELA units in V-basic (Figure 1A). On the LIA strips at time point t230, a stronger color reaction to OspA in V-plus was observed compared to V-basic (Figure 1B). While the antibody levels from the KELA decreased further in V-basic until the booster vaccination around t350 was applied, antibodies in dogs of V-plus increased after the additional booster on t180 and stayed at a significantly higher level until the yearly booster vaccination on t350 (Figure 1A). On t350, V-plus reached KELA units over 500, while in V-basic KELA units stayed below 300. The LIA results support the observations made with the KELA. The color reaction for OspA in V-basic was fainter than in V-plus and similar to the COC and was directly proportional to the decreasing antibody levels (Figure 1B). At the last observation time point (t390), antibodies of both groups displayed similar levels in the KELA (V-basic 588 KELA units on average; V-plus 610 KELA units on average; Figure 1A). LIA strips display a very strong signal for OspA in both groups on t390 after the last booster vaccination (Figure 1B).

**Figure 2:**
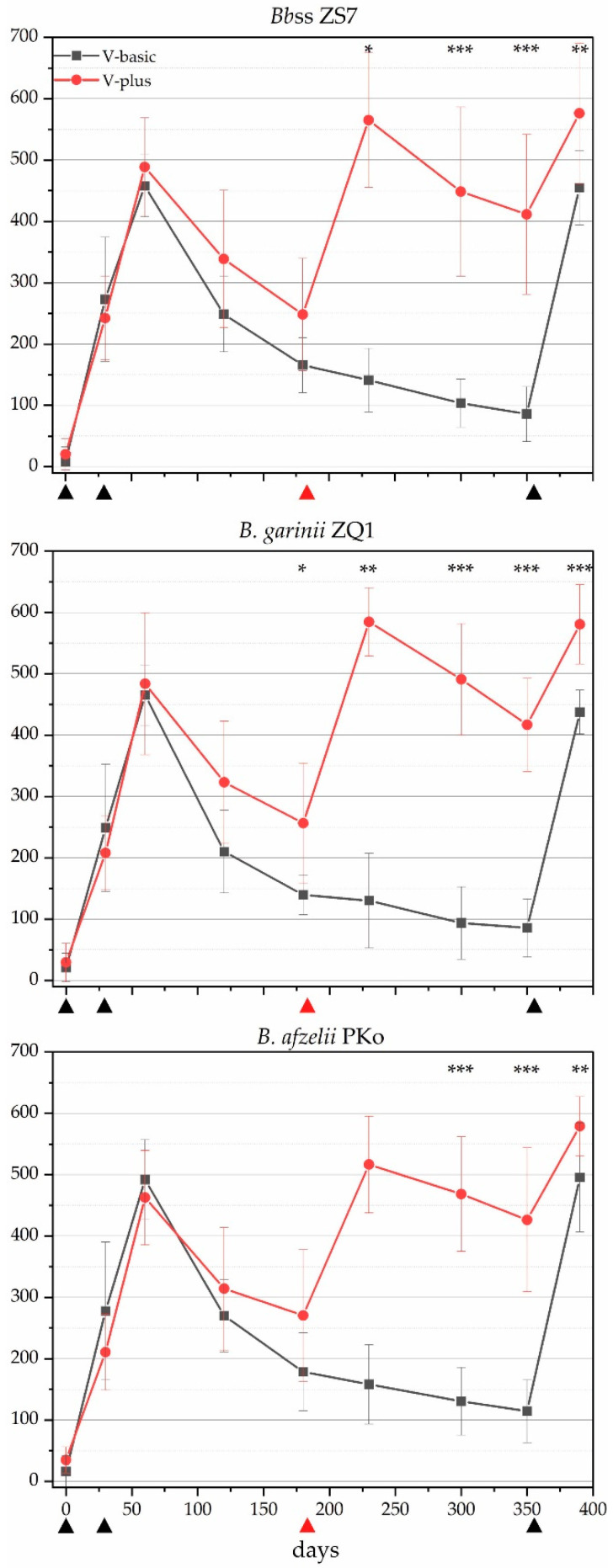
rOspA-specific antibody levels against *Borrelia* (*B.*) *burgdorferi* sensu stricto, *B. garinii,* and *B. afzelii* detected by the species-specific rOspA KELA. V-basic displayed as black squares; V-plus is shown as red circles. The whiskers depict the standard deviation on the specific time point. Vaccination time points are shown as black triangles in both groups; the red triangle indicates the additional booster vaccination in V-plus. * *p* ≤ 0.05; ** *p* ≤ 0.001; *** *p* ≤ 0.0001.

Reactions to species-specific recombinant OspA (rOspA) originating from the three *Bb*sl-complex species used in the lysate vaccine were subsequently analyzed by applying species-specific rOspA KELA assays (Figure 2). The antibody levels against *Bb*ss, *B. garinii*, and *B. afzelii* from both vaccination groups corresponded to the IgG KELA described above (Figure 1A). However, the measured levels for rOspA-specific antibodies for the three different borrelial species were lower than the IgG KELA levels (Figure 1A) for V-basic between t180 and t350. Further, rOspA KELA levels seem to be highly variable between different canine sera used in these assays. *Bb*ss-specific rOspA antibody levels in V-basic drop until t180 to a mean KELA level of 166 units and drop further until t350 to a mean KELA level of 86. On t180 mean KELA levels of 140 units were observed for *B. garinii*, that drop to 86 units on t350. Mean KELA levels for *B. afzelii* are at around 179 KELA units on t180 and declined to a mean of 115 KELA units on t350. When applying a booster vaccination on day 180 (i.e., V-plus), a drop in antibody levels until t180 was observed in this group as well. However, the booster vaccination on t180 lead to an increase in antibody levels on t230 (Figure 1). This increase led to higher antibody levels until the last booster vaccination on t350.

## 4. Discussion

Due to climate change and an increase in global temperature, we must expect an increase in tick population and a year-round activity of ticks [10,11,42]. This leads to the conclusion that there is a perennial risk of tick exposure and infection with TBD such as infections with *Bb*sl causing LB. As described above there are three columns of protection against LB: tick removal, use of repellents and acaricides, and vaccination. Tick removal only protects against LB when dogs are scanned for ticks after every walk and all ticks are found and removed promptly. Acaricides and repellents should be applied individually to the owner’s preferences and the dogs’ tolerance. All-year-around protection must be provided by reapplying the protective agent routinely. Further, ticks can develop resistance to these repellents and acaricides [27]. The vaccination against LB, based on inactivated *Bb*sl-complex species expressing the OspA provides the most reliable protection against LB, as the vaccination not only protects against the development of disease but further protects against the transmission of *Bb*sl-complex species to the canine host [43]. However, it is crucial to vaccinate against the genospecies that lead to infections in the dogs’ geographical environment and to provide perennial protection by keeping antibody levels high enough [44].

**Figure 3:**
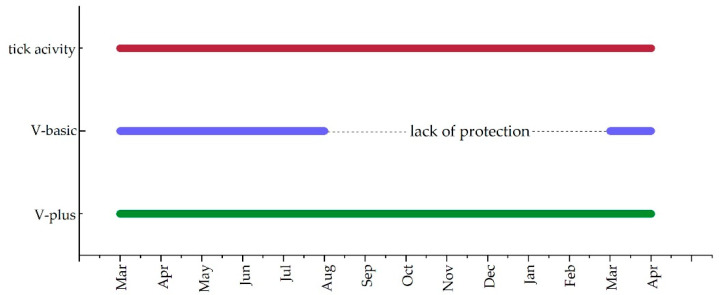
Tick activity and LB protection in different vaccination groups using two vaccination schemes compared over one year when LB vaccination is initiated in spring.

In this study, we hypothesized that the vaccination schedule of Merilym 3 (Boehringer Ingelheim Vetmedica GmbH, Ingelheim am Rhein, Germany) as provided by the manufacturer is not sufficient to protect dogs against LB, especially in autumn when a dog is immunized during the spring season. The basic vaccination is recommended to be applied at the beginning of tick season (i.e., spring) followed by a yearly booster vaccination. Other vaccines available in Germany (i.e., Virbagen canis B, Virbac Tierarzneimittel GmbH, Bad Oldesloe, Germany, and Rivac Borrelia, Ecuphar N.V., Oostkamp, Belgium) recommend a further booster vaccination four to six months after the basic immunization [45]. Therefore, we applied the vaccine Merilym 3 according to the vaccination scheme provided by the manufacturer and compared canine antibody levels of dogs receiving the standard vaccination scheme to antibody levels induced by a vaccination scheme with a booster vaccination on t180. Antibody levels were measured with a KELA system and vaccination-specific antibodies were further confirmed with a LIA. As Figure 1 depicts clearly, the standard vaccination scheme (i.e., V-basic) leads to a drop in antibody levels between t180 and t350. This can also be observed by the intensity of the signals on the LIA. When considering the antibody levels observed in the species-specific rOspA KELAs, it becomes very clear, that antibody levels induced with the standard vaccination scheme might not protect against borrelial transmission in autumn. Therefore, we conclude, that the standard vaccination scheme initiated in spring produces a gap in protection against LB in autumn (Figure 3).

Furthermore, the vaccination scheme with an additional booster on t180 is already recommended for the two other vaccines used in Germany [45]. Considering the decrease in antibody levels between t60 and t180, it might even be too late to apply the booster vaccination on t180 and as described for the other two German vaccines (i.e., Rivac Borrelia and Virbagen canis B) an earlier booster vaccination between days 100 and 150 might even be more adequate. Future research on this issue is urgently needed as well as the characterization of an antibody level protective against infection with borrelial organisms.

The authors would further like to address some general considerations regarding the LB vaccination in dogs. It has recently been discussed that the LB vaccine should still be considered a non-core vaccine in the vaccination guidelines [46] and applied according to the risk assessment of individual patients [45,47]. Therefore, veterinarians and veterinary clinics must be aware of the LB risk and apply individual recommendations regarding the dog’s exposure (i.e., working dogs, hunting dogs, traveling). Furthermore, the owners must be made aware of the risks of LB infection and thus must be informed about the importance of rigorous tick scanning after walks and the application of repellents and acaricides. Due to client failure in detecting and removing ticks and continuous application of repellents, adequate protection of canines might not be given and thus LB can occur. A safer method would be the continuous vaccination against LB in endemic regions. However, even when dogs are vaccinated against LB, owners need to keep in mind, that ticks transmit additional infectious agents like *Anaplasma* spp., *Ehrlichia* spp., which can only be avoided by removing ticks [23] and applying repellents or ectoparasitic agents. As tick-borne encephalitis virus is transmitted within an hour [48], avoiding an infection is only possible by applying repellents. Further, working and hunting dogs that due to their assignments are especially exposed to ticks and tick bites and thus endangered to infection with *Bb*sl-complex species should be protected against LB using every available option especially as their training is expensive and time-consuming and downtime due to LB can easily be avoided. The endemic regions are shifting due to climate change and the ability of ticks to reside in regions that have up to date not been habitable [1]. A further point that must be considered is the increase in tick population due to fewer population losses when winters are milder. Increased temperatures in winter, autumn, and spring further lead to increased activity of ticks in these seasons, which some owners might not be aware of. Additionally, rising global temperature and urban sprawl will increase the population of rodents and small mammals that will, in turn, contribute to the increase of tick populations in these areas [49]. In sum, veterinarians must be informed about such changes and the endemic regions of TBD and inform owners professionally and transparently of the best options for TBD prevention and LB protection, not only in the best interest of the dogs’ but also in the interest of the owners’ health.

Lately, the opinion has been expressed that the “rationale for canine LB vaccination is unpersuasive” [50] and the experts of the American College of Veterinary Internal Medicine could not agree on a recommendation for a LB vaccine [20]. This matter has further been reasoned with a statement of the American Animal Hospital Association regarding not recommended vaccines [51]. However, this reference refers to not recommended vaccines in pet cats and discusses the use of a vaccination against feline infectious peritonitis. Nevertheless, it is stated, that vaccines are not generally recommended for diseases with a low clinical significance, that display a good response to treatment, and where evidence of vaccination in the field is minimal and adverse events occur frequently [50]. Therefore, the authors feel obligated to address these issues as it affects the reputation of this important vaccine and harms the willingness of veterinarians to recommend and owners to make use of this vaccination. Regarding the low clinical significance, it has been discussed, that 95% of dogs display no clinical signs [50]. This statement first occurred in a study from 1992 by Levy et al. [19]. In this study, 234 dogs were analyzed for *Bb*sl-specific antibodies and according to their reaction divided into a seronegative (*n* = 109) and a seropositive (*n* = 125) group of dogs that were further monitored for 20 months. In both groups, the incidence of limb/joint disorder, lethargy, fever, and inappetence was nearly 5%. Retesting was possible in 202 canines (105 dogs from the seropositive group and 97 dogs from the seronegative group). Some of the examined canines from both groups were treated with antibiotics during the observation period. The observation of seropositive dogs seems incomprehensible, as we do not know when the infection occurred and how long it has been persisting. Regarding the seronegative group that seems sensible to observe, 88 from 97 dogs remained seronegative. Thus, nine dogs have seroconverted, of which two had received antibiotics. The clinical signs these nine dogs displayed are not clear. Further, the diagnosis of LB was based on the occurrence of limb/joint disorder accompanied by fever, lethargy, and inappetence [19]. As fever has been reported to only peak on single days [52] a co-occurrence would be observed rarely. Returning to the study by Levy et al. (1992) [19], a further sign of LB was the prompt response to antibiotic treatment. However, other bacteria might react to this antibiotic treatment as well, making the exclusion of differential diagnoses crucial. Regardless, differential diagnoses have not been ruled out. Serologic results have not been reported as the actual time of infection was unknown to the author. For animals diagnosed with LB due to clinical signs and prompt reaction to antibiotic treatment neither serologic results nor evidence of borrelial organisms have been reported (i.e., PCR, pathology, or serology) [19]. Therefore, the results from this study must be considered with caution. Nevertheless, this number of dogs displaying no clinical signs has first been cited by Appel et al. (1993) [12] and has in the following years been cited and used various times. However, this number of 5% clinically LB conspicuous dogs in the field is highly questionable and other researchers have provided numbers from clinical studies on dogs that are more reliable. Straubinger et al. (1997) [52] observed severe clinical lameness in 65% of canines experimentally infected and confirmed seropositivity, 10% of seropositive dogs displayed mild lameness, and 25% showed no clinical signs. Of canines that displayed clinical signs, 55% displayed an increase in temperature > 39.4 °C for a single day. In another study, 77% of dogs developed lameness, accompanied in 80% of lame dogs by concurrent fever (≥38.0 °C). In 92% of dogs, *Bb*ss organisms were recovered from skin and joint tissues samples [22]. In another trial, 69% of dogs developed clinically apparent arthritis after tick exposure [53]. Furthermore, described clinical signs of LB can vary strongly and canines displaying stiffness, lameness, arthritis, and joint swelling [52] are often not recognized and tested for LB. Additionally, further maladies like cardiac signs [54,55,56], nephritis [57,58,59], neurologic disorders [15,60], rheumatoid arthritis [61], and myositis [62] have been described to occur in canine patients with LB. It is questionable whether owners can connect these disorders with a tick bite that probably has occurred months before the onset of clinical signs [12,52] and if veterinarians then test dogs with these unspecific clinical signs for LB. Many dogs have been reported displaying no clinical signs [12,22,52], however, the authors wonder if we can recognize mild clinical signs and minimal to moderate pain in canines. In humans it is known that mild to moderate chronic pain affects physical and psychological health [63]. When considering a seroprevalence of around 2 to 20% [16,17], we have to assume that of 10.3 million dogs in Germany in 2021 [64] on average 11% (1,100,000 dogs) are infected with *Bb*sl-complex species. Considering that in animal trials on average 75% of dogs display clinical signs [52], we calculate that around 50% of dogs in the field might display clinical signs. Of the 1,100,000 infected dogs, at least around 550,000 dogs in Germany assumably might display clinical signs and probably experience a reduction in quality of life. Further, one needs to consider that the clinical signs of canines observed in trials have been conducted with *Bb*ss only [22,52]. However, other genospecies (i.e., *B. garinii* and *B. afzelii*) might lead to similar or further unrecognized disorders, therefore, research on clinical signs caused by these genospecies is necessary to evaluate the impact of *Bb*sl on dogs. In conclusion, in the authors’ opinion LB is no disease with low clinical significance and its well-documented appearance in endemic regions does not support the apprehension against LB vaccination.

Regarding the One Health concern, not only the transmission of antibiotic resistances but also the transmission of disease to humans and further animals must be considered. It has been implied that there is no direct transmission of *Bb*sl-complex species from animal to animal or from animal to human [50]. The authors agree with this statement and the absence of direct transmission has been proven in dogs [52]. However, many indirectly transmitted vector-borne diseases may be further dispersed by our pet animals and increase the risk of vector-exposition for humans. Infected nymphs or uninfected nymphs or larvae that can be infected during the blood meal on a seropositive dog may be carried into human habitats (i.e., urban parks or gardens), where the engorged nymphs then drop off and molt to adult ticks. These adults can then bite humans and thus transmit borrelial species [65]. A further concern is that vaccination of canines does not reduce LB prevalence [50]. In parts, the authors agree with this statement, as the vaccination of dogs does not influence the sylvatic infectious chain. However, considering that most dogs and humans will not go deep into the woods, and most humans living in cities will conduct their free-time activities in urban parks and greens, the carriage of ticks into these regions displays the greatest danger to the largest human population, townsfolk. Therefore, the authors consider an LB vaccination beneficial regarding the One Health concern.

Species distribution of borreliae is highly variable between the continents [3]. Therefore, it is not possible to use one single vaccine worldwide [37]. Vaccines used should contain all infective agents responsible for LB infection in the geographical area. For Europe, these agents are *B. afzelii*, *B. garinii*, and *Bb*ss [36]. All vaccines available in Germany are at least bivalent, however, the vaccine used in this study is the only trivalent option containing all three borrelial species. The vaccination, in combination with manual tick removal by the owners and the disciplined use of acaricides and repellents, allows the maximum of protection possible, as removal and repellents alone might not disable all tick attachment. In the authors’ opinion, the combination of these options with a vaccine is still the safest method to prevent LB in dogs. However, as ticks not only transmit LB, veterinarians and owners must be aware, that the vaccination is the best protection against LB, tick removal and repellents must still be conducted to protect canines against other TBD.

Retrospective observation originating from LB-vaccinated dogs show, that vaccination against LB is most effective when it is applied on days 0, 30, 180, and 360. When a booster vaccination on day 180 is missed, canines display a gap of immunity in autumn, while ticks still display a high questing activity. For the best protection in dogs, a booster vaccination on day 180 is urgently advised, as has been displayed for equines [39]. Further, the use of a canine LB vaccination in endemic regions, in general, seems highly advisable, especially regarding the One Health concern [65]. An infection with LB probably leads to the unnecessary suffering of many animals, that display clinical signs obvious or hidden to their owners, probably reducing the quality of life in these dogs. Infected dogs displaying clinical signs are treated with antibiotics that could have been renounced. Additionally, canines display a risk to their owners by carrying infected ticks in the urban space or by infecting uninfected ticks in the urban space [65].

## 5. Conclusions

The results from this retrospective study allow us to recommend a basic vaccination schedule with an additional booster vaccination on day 180 to ensure the best possible protection of dogs against Lyme borreliosis. Concerns regarding side effects seem unreasonable, as most vaccines in humans or dogs can display side effects, as do antibiotics that are necessary after infection. In this study, only 7% of vaccine injections led to side effects such as swelling or sensitivity at the injection site limited to hours or days. These temporally limited side effects cannot be compared to the clinical signs of LB and its’ possible chronic course. As such, there is an increasing need for veterinarians to inform dog owners about the importance of LB vaccination. The cost of a vaccination is affordable and side effects are neglectable compared to costs for diagnosis and therapy of LB, especially regarding the dogs’ well-being. In the future, it is strongly recommended to repeat this work with a higher number of canines and a longer observation period. The protection capacity of V-basic and V-plus throughout the year and especially the winter months might be an interesting research focus.

## Figures and Tables

**Table 1:** Allocation of time frames in the observational period in days and categorization to descriptive time points used in the further analysis.

Time Frame (Days)	Time Points	Available Serum Samples from V-basic	Available Serum Samples from V-plus
0	t0	28	26
25–50	t30	27	26
51–80	t60	27	26
100–135	t120	27	26
155–180	t180	25	26
185–265	t230	23	24
270–325	t300	22	24
325–369	t350	22	23
370–560	t390	21	22

V-basic, vaccinated on days t0, t30, and t350; V-plus, vaccinated on days t0, t30, t180, and t350.

## Data Availability

Not applicable.
